# KRAS acting through ERK signaling stabilizes PD-L1 via inhibiting autophagy pathway in intrahepatic cholangiocarcinoma

**DOI:** 10.1186/s12935-022-02550-w

**Published:** 2022-03-19

**Authors:** Zheng Gao, Jia-Feng Chen, Xiao-Gang Li, Ying-Hong Shi, Zheng Tang, Wei-Ren Liu, Xin Zhang, Ao Huang, Xuan-Ming Luo, Qiang Gao, Guo-Ming Shi, Ai-Wu Ke, Jian Zhou, Jia Fan, Xiu-Tao Fu, Zhen-Bin Ding

**Affiliations:** 1 Department of Liver Surgery and Transplantation, Liver Cancer Institute, Zhongshan Hospital, Fudan University, Shanghai, China; 2 Key Laboratory of Carcinogenesis and Cancer Invasion of Ministry of Education, Shanghai, China; 3 Shanghai Xuhui Central Hospital, Zhongshan-Xuhui Hospital, Fudan University, Shanghai, 200031 China; 4 Institutes of Biomedical Sciences, Fudan University, Shanghai, China

**Keywords:** KRAS, ERK signaling, Intrahepatic cholangiocarcinoma, PD-L1, Autophagy

## Abstract

**Background:**

While the correlation between PD-L1 expression and *KRAS* mutation has been previously reported in other solid tumors such as non-small cell lung cancer (NSCLC), whether PD-L1 can be modulated by ERK signaling downstream of KRAS in intrahepatic cholangiocarcinoma (iCCA) and the underlying molecular regulatory mechanism remain unclear.

**Methods:**

The expression of ERK, p-ERK, PD-L1 and autophagy markers following KRAS knockdown or Ras/Raf/MEK/ERK signaling inhibitors treatment was examined in two human iCCA cell lines (HuCCT1 and RBE) using western blotting and immunofluorescence. Both pharmacological autophagy inhibitors and short-interfering RNA against *ATG7* were applied to inhibit autophagy. The apoptosis rates of iCCA cell lines were detected by flow cytometry and CCK-8 after co-culturing with CD3/CD28-activated human CD8^+^ T lymphocytes. Immunohistochemistry was applied to detect the correlation of ERK, p-ERK and PD-L1 in 92 iCCA tissues.

**Results:**

The present study demonstrated that the PD-L1 expression level was distinctly reduced in *KRAS*-mutated iCCA cell lines when ERK signaling was inhibited and ERK phosphorylation levels were lowered. The positive association between p-ERK and PD-L1 was also verified in 92 iCCA tissue samples. Moreover, ERK inhibition induced autophagy activation. Both inhibiting autophagy via autophagy inhibitors and genetically silencing the *ATG7* expression partially reversed the reduced PD-L1 expression caused by ERK inhibition. In addition, ERK-mediated down-regulation of PD-L1 via autophagy pathways induced the apoptosis of iCCA cells when co-cultured with CD3/CD28-activated human CD8^+^ T lymphocytes in vitro.

**Conclusions:**

Our results suggest that ERK signaling inhibition contributes to the reduction of PD-L1 expression through the autophagy pathway in iCCA. As a supplement to anti-PD-1/PD-L1 immunotherapy, ERK-targeted therapy may serve as a potentially novel treatment strategy for human *KRAS*-mutated iCCA.

## Introduction

Cholangiocarcinoma (CCA) is the second most common primary liver malignancy that accounts for approximately 15% of cases of liver cancers and ~ 3% of all gastro-intestinal malignancies [1, 2]. In recent years, the morbidity and mortality of CCA have been steadily rising. Anatomically, CCA can be classified as intrahepatic (iCCA), perihilar, or distal cholangiocarcinoma [3]. There are limited treatment options for CCA, which is a lethal malignancy with a five-year overall survival (OS) rate of only ~ 10% and a median OS of ~ 24 months [4]. Currently, surgical resection and liver transplantation are the only curative treatment approaches used in early-stage patients. However, once diagnosed, the iCCA patients are generally in the advanced stages of the disease when an effective surgical treatment is no longer possible. Moreover, present therapeutic strategies have a limited efficacy. Therefore, effective therapeutic strategies and potential molecular mechanisms to combat CCA are necessary.

Numerous studies have unveiled *KRAS, BRAF, IDH1/2, FGFR2*, and *EGFR* as prevalent oncogenic drivers in CCA [5], especially the *KRAS* mutations, which might be one of the most frequent molecular alterations in iCCA [6]. Once KRAS is activated, the Raf/MEK/ERK pathway is hyper-activated, thus, modulating multiple cellular processes, including survival, proliferation, and differentiation. Therefore, plentiful research studies have been dedicated to study this pathway’s inhibition, including using MEK1/2 and ERK1/2 inhibitors [7–9]. Even though MEK inhibitors, such as trametinib, have been extensively investigated, whether ERK inhibitors can be used for the treatment of iCCA, especially for *KRAS*-mutated iCCA, has not been properly evaluated in vivo or in vitro.

A wide spectrum of cancers, including iCCA, have been treated using the immune checkpoint blockade (ICB) therapy. As the most crucial immune checkpoint, the PD-L1 expression level determines the efficacy of anti-PD-1/PD-L1 immunotherapy. Accumulating evidence has suggested that KRAS can regulate PD-L1 expression in other cancers, such as NSCLC [10]. However, whether PD-L1 can be modulated by ERK in iCCA and the underlying molecular mechanisms remain unclear. In addition, as a highly conserved intracellular material degradation process, autophagy is involved in immune responses and cell homeostasis maintenance [11]. A recent study has discovered that autophagy regulates PD-L1 expression in gastric cancer [12]. Consequently, the link between ERK signaling, PD-L1, and autophagy in iCCA needs to be determined.

The present study uncovered an underlying molecular mechanism for PD-L1 regulation by ERK signaling via autophagy activation and provided a novel immune-related therapeutic strategy that regulates the ERK signaling pathway.

## Materials and methods

### Cell lines and cell culture

Human iCCA cell lines HuCCT1 and RBE were purchased from the Cell Bank of Type Culture Collection of Chinese Academy of Sciences (Shanghai, China) and maintained in RPMI 1640 medium (Sigma-Aldrich, MO, USA) supplemented with 10% heat-inactivated fetal bovine serum (Gibco, USA), 100 U/mL penicillin, and 100 mg/mL streptomycin (Sigma-Aldrich). All cell lines were cultured at 37 °C in a humidified incubator with 5% CO_2_.

### Antibodies and reagents

The antibodies used in the present study included the following: anti-SQSTM1/p62 rabbit mAb (ab109012) from Abcam, anti-LC3A/B (12741), anti-ATG7 (8558), anti-PD-L1 (13684 or 41726), anti-KRAS (3339), anti-p44/42 MAPK (Erk1/2) (4695), anti-Beclin-1(3495), anti-ATG5 (12994), and anti-phospho-p44/42 MAPK (Erk1/2) (Thr202/Tyr204) (4370) from Cell Signaling Technology, and anti-GAPDH from Yeasen. Annexin V-APC and 7-AAD apoptosis detection kit were purchased from Biolegend. Trametinib and SCH772984 were purchased from Selleck Chemicals (Houston, TX, USA).

### Western blotting analysis

Western blotting analysis was performed as previously described [13]. Briefly, the proteins from total cell lysates were separated using 10% or 12.5% standard sodium dodecyl sulfate polyacrylamide gels and then transferred to polyvinylidene difluoride membranes. The membranes were washed, blocked, and incubated at 4 °C overnight with specific primary antibodies against Beclin-1, ATG5, ATG7, LC3, ERK, p-ERK, KRAS or PD-L1 (1:1000), P62(1:7000), and GAPDH (1:5000), followed by incubation with horseradish peroxidase (HRP)–conjugated secondary antibodies. The intensity of protein bands was determined using densitometry with a Bio-Rad system (Bio-Rad Laboratories, CA, USA).

### RNA interference

Three different sequences targeted to three different sites in *KRAS* and *PD-L1* were designed by Genechem (Shanghai, China). Sense and antisense strands for siRNAs were as follows: si*KRAS*-1: 5’-CGAAUAUGAUCCAACAAUATT-3’; 5’-UAUUGUUGGAUCAUAUUCGTT-3’; si*KRAS*-2: 5’-AGCAAGAAGUUAUGGAAUUTT-3’;5’-AAUUCCAUAACUUCUUGC-UTT-3’; si*KRAS*-2: 5’-CAAGAGGAGUACAGUGCAATT-3’; 5’-UUGCACUGUACUC-CUCUUGTT-3’; siPD-L1: 5’-GACCUAUAUGUGGUAGAGUAU-3’;5’-AUACUCUACCACAUAUAGGUC-3’; *KRAS* and *PD-L1*siRNAs were transfected into iCCA cells using Lipofectamine 3000 (Invitrogen, USA). Cells were lysed 72 h after transfection, and proteins levels were assayed using western blotting analysis.

### Tissue microarray (TMA) and immunohistochemistry analysis

TMA was constructed as previously described [14]. In summary, all patients diagnosed with hepatocellular carcinoma were reviewed by two histopathologists, and representative areas were pre-marked in the paraffin blocks away from necrotic and hemorrhagic materials. Duplicates of 1-mm-diameter core biopsies from tumor center and para-tumoral noncancerous area were taken from the donor tumor tissues and transferred to the defined array positions. Thus, a TMA block containing 184 cylinders was constructed (Shanghai Biochip Co., Ltd.). Sections (4 μm in thickness) were placed on 3-aminopropyltriethoxysilane–coated slides. Immunohistochemistry was performed with monoclonal rabbit antibodies against human ERK (1:250), p-ERK (1:400), and PD-L1 (1:200) using 4-μm-thick paraffin-embedded tumor tissue sections excised from patients diagnosed with iCCA at the Liver Cancer Institute, Zhongshan Hospital, Fudan University, Shanghai, China. After deparaffinization and rehydration, antigen retrieval was performed using pepsin (10 μM) for 20 min at 37 °C and by boiling the slides in citrate buffer (pH 6.0) for 10 min in a microwave for all other antigens. Cooled slides were washed in phosphate-buffered saline (PBS; 3 × 15 min each) and incubated in 3% hydrogen peroxide for 10 min to block endogenous peroxidase activity. Following washing, slides were blocked with 10% goat serum for 1 h at room temperature and probed with primary antibodies overnight at 4 °C in a humid chamber. Slides were then incubated with HRP-conjugated secondary antibodies for 1 h at room temperature. After washing, slides were developed using a diaminobenzidine substrate kit (Gene Tech, Shanghai, China), counterstained with hematoxylin for 2 min, washed, and dehydrated.

### Patients and specimens

Patient samples were collected after obtaining informed consent from each patient according to an established protocol approved by the Ethics Committee of Zhongshan Hospital. The data did not contain any information that could lead to identification of individual patients.

Tumor specimens for TMA studies were obtained from 92 consecutive iCCA patients who underwent curative resection without preoperative treatment at the Liver Cancer Institute, Zhongshan Hospital, Fudan University. Samples were collected immediately after resection, transported in liquid nitrogen, and stored at −80 ℃. An additional 11 fresh tissue samples were also obtained for western blotting.

### Flow cytometry

Activated T cells were added into the co-culture system with HuCCT1 or RBE cells treated with CQ or transfected with shATG7 at a ratio of 1:1, respectively. After 24 h, suspended T cells and the trypsinized HuCCT1 or RBE cells were removed from the cell culture plate. Then, after washing with PBS, the suspended cells were stained with Annexin V-APC and 7-AAD for 15 min with an apoptosis detection kit at room temperature (Biolegend, San Diego, CA, USA). Finally, the apoptotic cells were evaluated using flow cytometry (BD FACSAriaTM II, NJ, USA) and the data were analyzed using FlowJo software according to the manufacturer’s instructions.

### Immunofluorescence and confocal microscopy

For immunofluorescence analysis, cells were fixed with 4% paraformaldehyde for 15 min, permeabilized in PBS containing 0.1% Triton X-100 for 10 min, and then blocked with 5% bovine serum albumin for 20 min. Slides were incubated with the indicated primary antibodies overnight at 4 °C, followed by incubation with Alexa Fluor 488 secondary antibody for 1 h at room temperature. Then, 4’,6-diamidino-2-phenylindole was used to stain the nuclei and images were acquired using confocal microscopy (Olympus FV-3000, Japan).

### Cell counting kit-8 assay

Cellular apoptosis rate was detected using a cell counting kit-8 **(**CCK-8) (Dojindo, Japan) according to the manufacturer’s instructions. The indicated cells were inoculated into 96-well plates. At the indicated point, 10 μL of the CCK-8 solution were added into each well with 100 µL of culture media. The absorbance of each individual well was measured at 450 nm.

### T cell-mediated killing assay

Peripheral blood mononuclear cells isolated from heparinized peripheral blood samples were obtained from healthy donors using Ficoll-Paque (GE Healthcare Life Sciences) density gradient centrifugation. They were cultured in ImmunoCult™-XF T Cell Expansion Medium (STEMCELL Technologies, Canada) with ImmunoCult Human CD3/CD28 T cell activator (STE-MCELL Technologies) and IL-2 (600 U/mL; PeproTech, USA) for one week according to the manufacturer’s protocol. Cancer cells were seeded in the plates overnight and then incubated with CQ (5 or 10 μM) for 24 h, followed by incubation with activated T cells for 24 h. The ratio between cancer cells and activated T cells was 1:1. T cells and cell debris were removed using a PBS wash and the remaining cells were stained with crystal violet or used in subsequent experiments.

### Statistical analysis

Differences between two groups were evaluated using paired or unpaired two-tailed Student's t-test, while multiple groups were analyzed using one-way analysis of variance. Correlation analyses were used to analyze the relationships among the expression levels of ERK, pERK, and PD-L1. Representative results from three independent experiments were used in the study analysis. Statistical significance was set at P < 0.05. All analyses were performed using SPSS version 26.0 software (IBM SPSS Inc., USA).

## Results

### *KRAS* knockdown inhibits PD-L1 expression

**Fig. 1:**
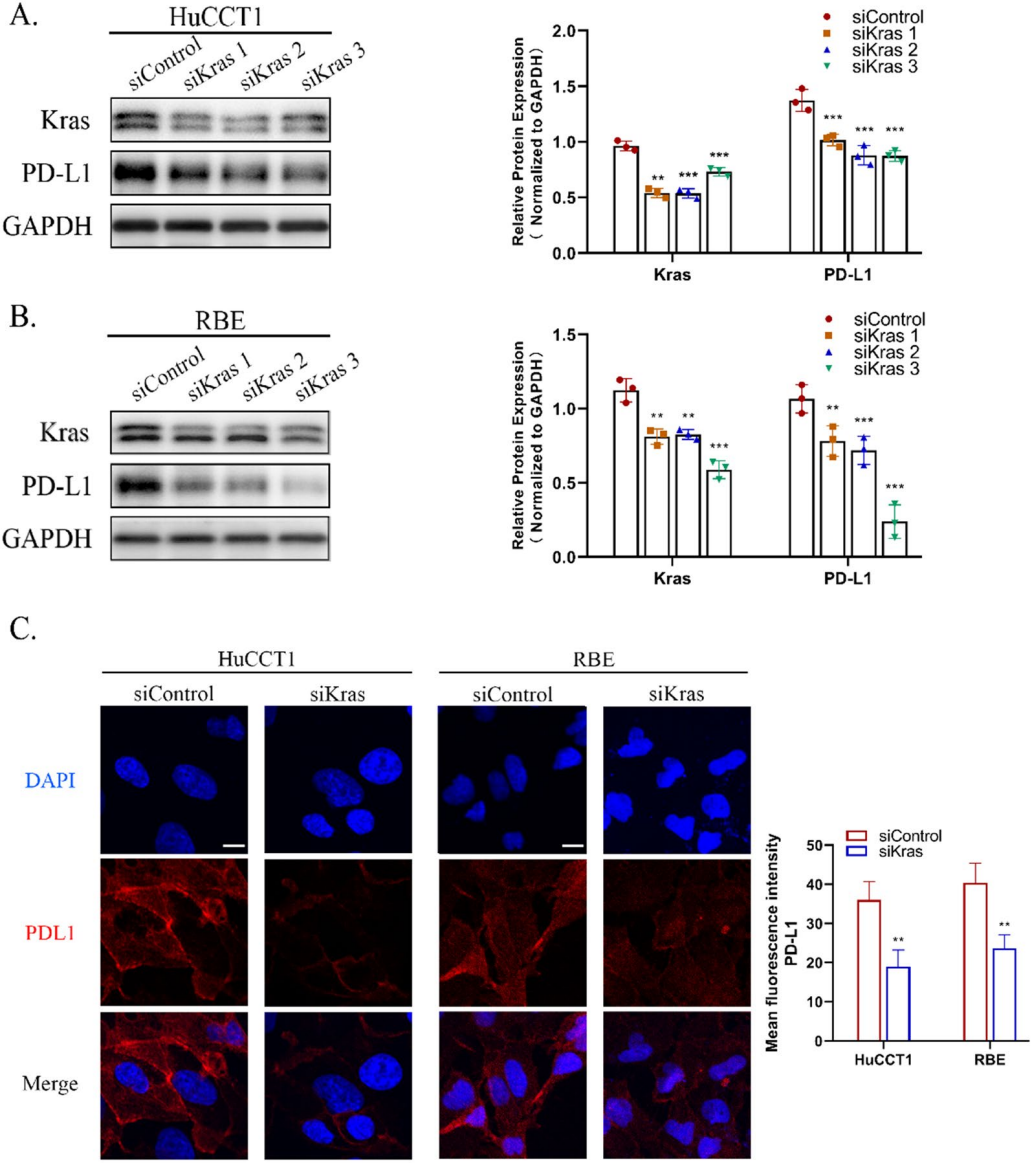
Knockdown of *KRAS* inhibited the expression of PD-L1. **A–B** Western blotting analysis of KRAS and PD-L1 in HuCCT1 and RBE cell lines after treatment with siRNA targeting *KRAS*. **C** Representative images of positive staining of PD-L1 (red) and DAPI (blue) was determined by immunofluorescence using confocal microscopy in HuCCT1 and RBE cells treated by siRNA knockdown (Scale bar, 150 μm)

In order to investigate whether oncogenic KRAS regulates the expression of PD-L1 following *KRAS* knockdown, it was examined using western blotting in two human iCCA cell lines (HuCCT1 and RBE) with endogenous *KRAS* mutations [15] (Fig. 1A, B). Intriguingly, the PD-L1 protein level was significantly reduced in *KRAS* knockdown cells. Immunofluorescence staining results also revealed that *KRAS* knockdown caused a significant reduction in PD-L1 expression. PD-L1 (red fluorescence) showed a stronger membrane localization in normal cells compared to *KRAS* knockdown cells (Fig. 1C). Taken together, these results demonstrated that knocking down *KRAS* reduced the PD-L1 expression level, which was also correlated with *KRAS* mutation.

### *KRAS* knockdown down-regulates PD-L1 via ERK signaling

**Fig. 2:**
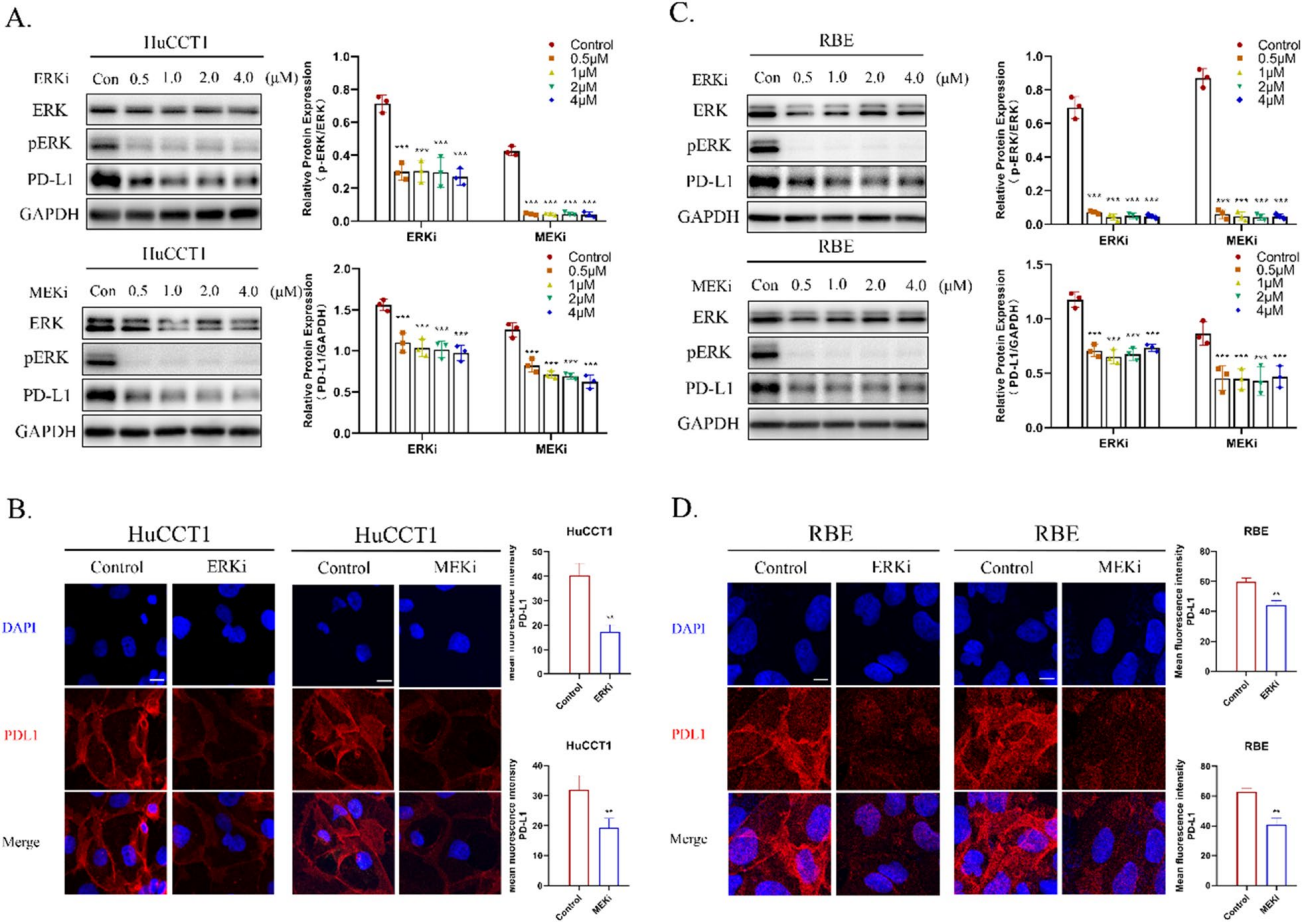
Effects of ERK inhibitors or MEK inhibitors on expression of PD-L1 in iCCA cell lines. **A**, **C** Levels of ERK, p-ERK and PD-L1 were validated by western blotting in HuCCT1 and RBE cells treated by different concentrations (0.5, 1.0, 2.0, 4.0 μM) of ERKi or MEKi for 24 h. **B**, **D** Representative images of positive staining of PD-L1 (red) and DAPI (blue) was determined by immunofluorescence using confocal microscopy in HuCCT1 and RBE cells treated by ERKi (4 μM) or MEKi (4 μM) for 24 h (Scale bar, 150 μm)

Although the role of PD-L1 in ICB therapy has been well established [16], the regulatory mechanism of its expression by *KRAS* mutations remains poorly understood. Based on the recent reports that *KRAS* mutation stimulates PD-L1 expression via the ERK signaling pathway [17], we postulated that ERK inhibition might contribute to the decreased PD-L1 expression. To validate this hypothesis, HuCCT1 and RBE cells were treated with different concentrations of Ras/Raf/MEK/ERK signaling inhibitors for 24 h, including trametinib (MEK1/2 inhibitor) and SCH772984 (ERK1/2 inhibitor). PD-L1 protein level in HuCCT1 (Fig. 2A) and RBE (Fig. 2C) cells treated with trametinib or SCH772984 was obtained using western blotting. Both MEK1/2 and ERK1/2 inhibitors reduced the expression of p-ERK, which further resulted in down-regulation of PD-L1 expression (Fig. 2A, C). Consistently, immunofluorescent staining analysis also verified the decreased expression of PD-L1 (red fluorescence) on the cytomembrane of HuCCT1 and RBE cells following trametinib or SCH772984 treatment (Fig. 2B, D). These results indicated that *KRAS*-mutated iCCA cells induced the expression of PD-L1 via the p-ERK signaling pathway.

### Pharmacological inhibition of ERK1/2 promotes PD-L1 degradation via autophagy induction

**Fig. 3:**
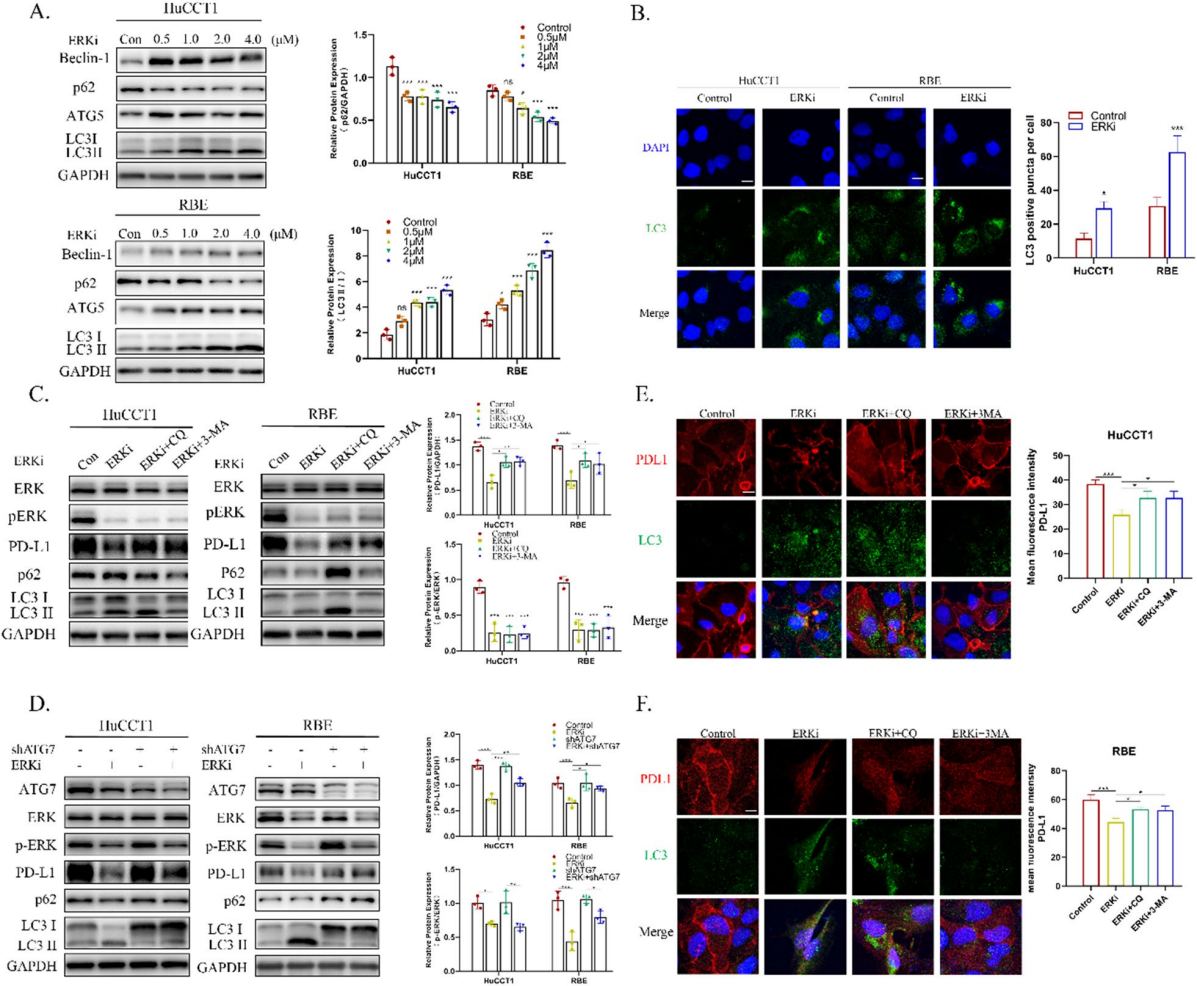
Pharmacological inhibition of ERK1/2 promotes PD-L1 degradation via autophagy induction. **A** ERK inhibition by ERKi in different concentrations (0.5, 1.0, 2.0, 4.0 μM) in HuCCT1 and RBE cells induced the conversion of LC3-I to LC3-II. **B** Representative images of positive staining of LC3 (green) and DAPI (blue) was determined by immunofluorescence in HuCCT1 and RBE cells treated by ERKi (4 μM) for 24 h. (Scar bar, 20 μm). **C** Western blotting analysis of ERK, p-ERK, PD-L1, p62/SQSTMQ1 and LC3-I/II after the treatment of ERKi alone or a combination of ERKi and autophagy inhibitors, including chloroquine (CQ, 5 μM) or 3-methyladenine (3-MA, 5 μM) in HuCCT1 and RBE cells for 24 h. **D** Western blotting analysis of ERK, p-ERK, PD-L1, p62/SQSTMQ1 and LC3-I/II upon the autophagy inhibiting by knocking down *ATG7* in HuCCT1 and RBE cells with or without ERKi pretreatment. The knockdown efficacies of *ATG7* were verified. The conversion of LC3-I to LC3-II was reduced. **E**–**F** Representative images of positive staining of PD-L1 (red), LC3-II (green) and DAPI (blue) was determined by immunofluorescence after treatment of ERKi alone or a combination of ERKi and CQ(5 μM) or 3-MA(5 μM) in HuCCT1 and RBE cells for 24 h (Scale bar, 150 μm)

It has been reported that inhibition of Ras/Raf/MEK/ERK signaling can elicit autophagy [18]. Simultaneously, autophagy, which is a lysosome-mediated process of cellular recycling, might be involved in the ERK inhibition-mediated degradation of PD-L1. To verify this hypothesis, HuCCT1 and RBE cells were treated with different concentrations of SCH772984 for 24 h. Consistent with our assumption, the conversion of endogenous LC3-I to the lipidated, autophagosome-associated form of LC3-II, the upregulation of Beclin-1 and ATG5 protein level, and a decrease in p62/SQSTM1 protein level were observed, which indicated an induction of autophagy (Fig. 3A, B). At the same time, PD-L1 expression was reduced (Fig. 2A, C). To further assess if PD-L1 degradation was autophagy-mediated, HuCCT1 and RBE cells were treated with either SCH772984 alone or a combination of SCH772984 and autophagy inhibitors, including chloroquine (CQ, inhibiting autophagy through increasing the lysosomal pH so that impairs lysosomal function) or 3-methyladenine (3-MA, blocking autophagy through inhibiting the class III PtdIns3K). SCH772984 co-treatment with autophagy inhibitors for 24 h resulted in a PD-L1 protein level recovery compared to the groups treated with SCH772984 alone (Fig. 3C). In addition to the pharmacological autophagy inhibition, the essential autophagy-related gene *ATG7* involved in the formation of autophagosome was suppressed by shRNA. Similar to the effect of pharmacological autophagy inhibitors, *ATG7* knockdown significantly reversed the reduced PD-L1 expression (Fig. 3D) caused by the ERK inhibitor (ERKi), which further indicated the autophagic degradation of PD-L1. Furthermore, confocal microscopy results showed that ERK inhibition produced increased LC3 puncta and induced its co-localization with PD-L1, while inhibiting autophagy reversed PD-L1 expression (Fig. 3E, F). Therefore, autophagy is part of the mechanism by which ERK inhibition leads to the intracellular degradation of PD-L1.

### ERK1/2 inhibition enhances apoptosis of iCCA cells in co-culture system

**Fig. 4:**
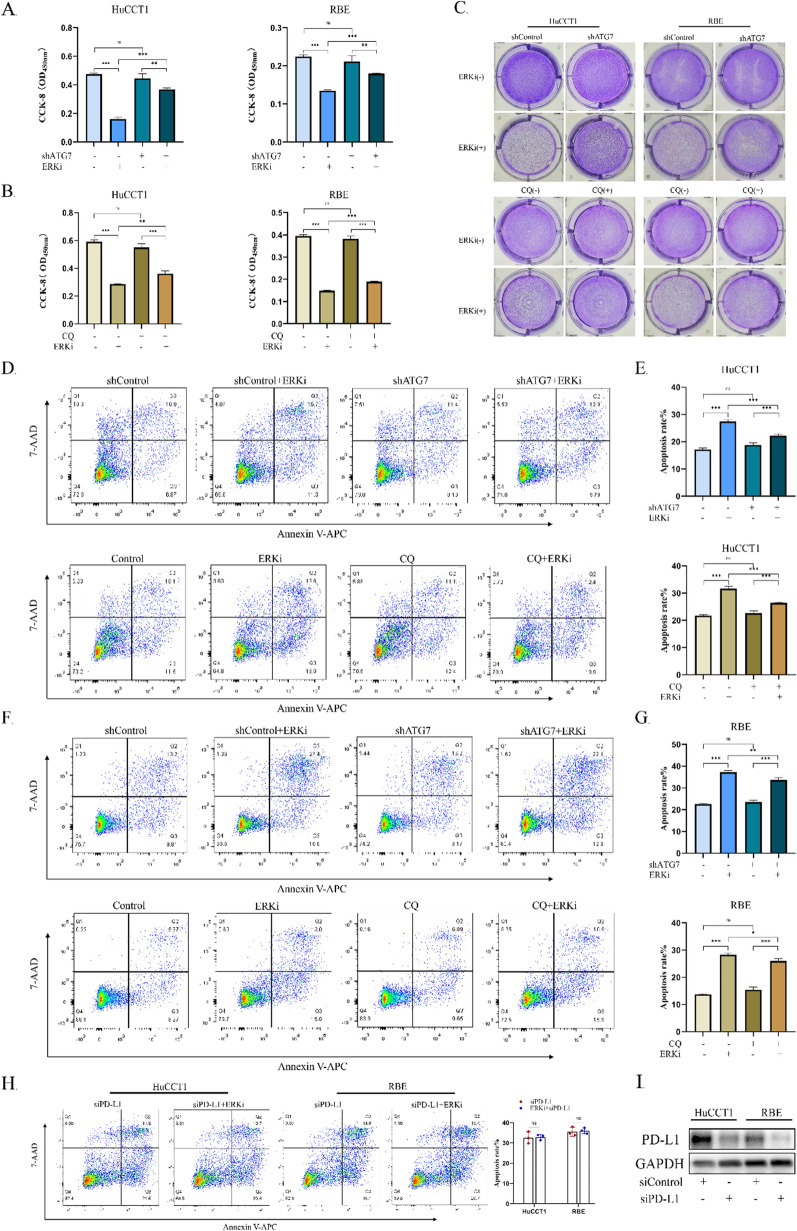
ERK1/2 inhibition enhances apoptosis of iCCA cells in co-culture system. **A** Pretreated with ERKi(4 μM), the viability of HuCCT1 and RBE cells with knockdown of *ATG7* was detected by CCK-8 assay after co-culturing with activated CD8^+^T cells for 24 h. **B** Pretreated with ERKi(4 μM) alone or a combination of ERKi and CQ(5 μM), the viability of HuCCT1 and RBE cells was detected by CCK-8 assay after co-culturing with activated CD8^+^T cells for 24 h. **C** Pretreated with ERKi (4 μM) alone, a combination of ERKi(4 μM) and CQ (5 μM), or knockdown of *ATG7*, HuCCT1 and RBE cells co-cultured with activated T cells for 24 h were subjected to crystal violet staining. **D**, **F** The apoptosis rates of HuCCT1 or RBE cells were detected by Annexin V-APC/7-AAD apoptosis assay using flow cytometry in iCCA cells and activated CD8^+^T cells co-culture system. **E**, **G** Fig. E and G are the statistical histogram of **D** and **F**. **H** The apoptosis rates of HuCCT1 or RBE cells after PD-L1 knockdown alone or a combination of ERKi treatment and PD-L1 knockdown were detected by Annexin V-APC/7-AAD apoptosis assay using flow cytometry in co-culture system. Ns, not significant. **I** PD-L1 protein expression was analyzed by western blot upon PD-L1 knockdown using siRNA. Data shown are mean (SD) from three independent experiments. Ns, not significant. *P < 0.05.**P < 0.01.***P < 0.001

To investigate whether down-regulation of PD-L1 mediated by ERK inhibition enhances apoptosis in HuCCT1 and RBE cells via cytotoxic T lymphocytes, human CD8^+^ T lymphocytes were prepared from peripheral blood donated by healthy volunteers and co-cultured with HuCCT1 and RBE cells at a ratio of 1:1. The experimental results suggested that the apoptosis rates of HuCCT1 and RBE cells pretreated with ERK inhibitor were higher compared to those of the control group after co-culturing with CD3/CD28-activated human CD8^+^ T lymphocytes (Fig. 4A–C). In addition, to investigate whether autophagy inhibition restricts the apoptosis of iCCA cells via a recovery of PD-L1 expression, a co-culture system was established between autophagy-inhibited HuCCT1 or RBE cells and CD8^+^ T lymphocytes. As expected, either knockdown of the autophagy-related gene *ATG7* (Fig. 4A) or pharmacological autophagy inhibition (Fig. 4B) reduced the apoptosis rates of HuCCT1 and RBE cells in the co-culture system. Similar to the above results, flow cytometry analysis confirmed that ERK inhibition enhanced apoptosis of HuCCT1 (Fig. 4D, E) and RBE (Fig. 4F, G) cells, while autophagy defects in both of them significantly reduced the percentage of apoptotic tumor cells when co-cultured with activated CD8^+^T lymphocytes. Furthermore, we validated whether ERK inhibition enhances cancer cell apoptosis through PD-L1 in co-culture system. The flow cytometry analysis showed that upon PD-L1 knockdown (Fig. 4I), the apoptosis rates of HuCCT1 and RBE cells following ERK inhibitor treatment did not change significantly compared with the group treated with PD-L1 knockdown alone (Fig. 4H), which was consistent with our hypothesis. These results demonstrated that ERK inhibition-mediated down-regulation of PD-L1 induces the apoptosis of iCCA cells, while blocking autophagy flux reverses PD-L1 expression, which further alleviates iCCA cell apoptosis.

### PD‑L1 expression is correlated with p-ERK in patients with iCCA

**Fig. 5:**
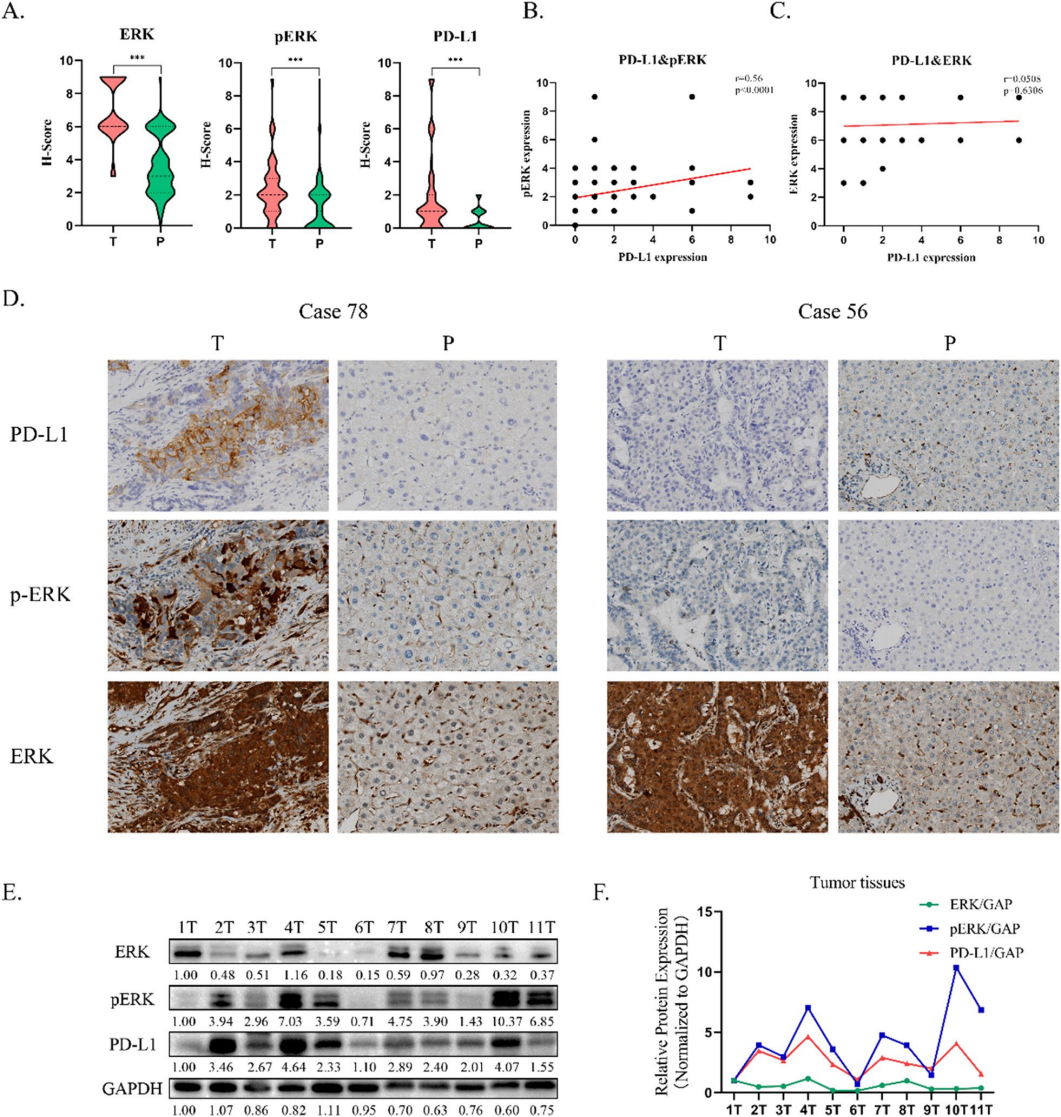
PD‑L1 expression is correlated with p-ERK in patients with iCCA. **A** The expression levels of ERK, p-ERK and PD-L1 between tumor tissues and para-tumor tissues based upon IHC expression score. **B** p-ERK levels were positively correlated with the levels of PD-L1 protein based upon IHC expression score. Spearman r = 0.56. P < 0.0001. **C** ERK levels were not correlated with the levels of PD-L1 protein based upon IHC expression score. Spearman r = 0.0508. P = 0.6306. **D** Representative immunohistochemical staining of PD-L1, p-ERK and ERK in tumor tissues and para-tumor tissues of different patients. **E** The protein levels of ERK, p-ERK and PD-L1 were detected by western blotting analysis of cell lysates isolated from an additional 11 tumor tissues. GAPDH was used as loading control. **F** The relative protein expression (Normalized to GAPDH) of ERK, p-ERK and PD-L1 in iCCA tissues. Magnification, ×100. ***P < 0.001

To validate the finding that ERK inhibition decreases the PD-L1 expression level, ERK, p-ERK, and PD-L1 expression was analyzed using immunohistochemistry in patients’ iCCA tissue samples. ERK and p-ERK were mainly expressed in the cytoplasm of cancer cells, while PD-L1 demonstrated a more pronounced membrane localization (Fig. 5D). Compared to the para-tumor tissues, ERK, p-ERK, and PD-L1 expression was significantly higher in tumor tissue samples (Fig. 5A). Among 92 patients, p-ERK expression in 46 (50%) patients and ERK expression in almost all patients was positive. With regard to PD-L1 expression, 31 cases demonstrated a positive PD-L1 staining and 61 cases showed a negative PD-L1 staining. More importantly, patients with a low expression of p-ERK tended to have a weaker intensity of PD-L1 staining than patients with a high expression of p-ERK regardless of ERK expression level (Fig. 5D). Based on the immunohistochemistry score, a positive correlation between PD-L1 and p-ERK expression levels (Fig. 5B) was revealed using Spearman’s correlation analysis (PD-L1 vs. p-ERK, P < 0.0001, Fig. 5B; PD-L1 vs. ERK, P = 0.6306, Fig. 5C). Furthermore, to confirm these observations, levels of ERK, p-ERK, and PD-L1 proteins isolated from an additional 11 tumor tissues were analyzed using western blotting (Fig. 5E). Consistent with the immunohistochemistry results, PD-L1 protein level was positively associated with p-ERK expression rather than ERK expression (Fig. 5F).

## Discussion

A high prevalence of oncogene *KRAS* mutations have been frequently observed in a variety of cancers, including human iCCA [19, 20]. In general, KRAS gene mutation at different prevalence rates is associated with a series of highly fatal cancers. The top 5 human cancers among these KRAS somatic mutations are pancreas (57%), large intestine (35%), biliary tract (28%), small intestine (17%) and lung (16%) [21]. Apparently, the mutation sites and frequencies of KRAS gene were varied from diverse human tumor types. For example, three amino acid residues with missense mutations including G12(codon 12), G13(codon 13), and Q61(codon 61) are the most common mutation sites with distinct mutation frequencies in different cancers [22]. Among these mutations, point mutations in KRAS codon 12 are probably the most common event, which accounts for approximately 80% of KRAS mutation [21]. A recent study also showed that, compared with a western iCCA cohort (the Memorial Sloan Kettering Cancer Center cohort), the China Fudan-iCCA cohort showed higher KRAS mutation frequency and lower IDH1, ARID1A, and TERT mutation frequencies [23]. Most studies have concluded that there is a negative influence on prognosis of patients with a *KRAS* mutation whether or not they have undergone surgery for other solid tumors [24, 25] and a correlation between oncogenic *KRAS* mutations and PD-L1 expression in cancers has also been emphasized [10, 26, 27]. As a major downstream part of KRAS signaling, the Ras/Raf/MEK/ERK pathway plays a pivotal role in tumor initiation, progression, and differentiation. Nevertheless, the mechanism of KRAS with its downstream pathways in modulating PD-L1 expression remained uncertain. The present study suggested that RNA interference in *KRAS* down-regulates the expression of PD-L1 via ERK signaling in an autophagy-dependent manner as illustrated above. Based on this foundation, it was speculated that the poor prognosis of *KRAS*-mutated patients is due to the continuous activation of the Ras/Raf/MEK/ERK pathway caused by the *KRAS* gene mutation, which might lead to up-regulation of PD-L1 in tumor cells by abrogating autophagic degradation and further promoting the immune escape.

To investigate the effects of ERK inhibition on PD-L1 expression in *KRAS*-mutated iCCA cells, HuCCT1 and RBE cells were treated with MEKi and ERKi for 24 h. The results demonstrated that the inhibition of MEK by trametinib and ERK by SCH772984 promoted the degradation of PD-L1, but not in a dose-dependent manner. In addition, these results showed that there was little change in the expression of ERK after using ERKi, while ERK phosphorylation and PD-L1 level were altered significantly, suggesting that it was functional phosphorylated ERK that modulated the expression of PD-L1. Immunohistochemical score and western blotting results also showed a positive association between p-ERK and PD-L1 (Fig. 5B, D and E). In line with the present findings, mounting evidence has pointed to a key role of ERK signaling in modulating the expression of PD-L1. Upon activation, ERK signaling reinforced the binding of c-JUN and PD-L1 promoters, which further recruited STAT3 to increase PD-L1 expression in NSCLC [28]. Furthermore, it has also been reported that PD-L1 expression induced by TRAIL was dependent on the p-ERK/STAT3 signaling pathway in esophageal squamous carcinoma [29]. Except for ERK signaling, the PI3K/AKT, JAK/STAT3, and Wnt/β-catenin signaling pathways were also involved in the modulation of PD-L1 expression in various solid tumors [30–32]. In general, these studies and the present results have demonstrated that it was the ERK signaling pathway, or more precisely the functional p-ERK, that mainly modulated the expression of PD-L1.

Previous studies have reported that trametinib promotes the autophagic flux in RAS-driven cancers [18]. As the downstream component of the Ras pathway, it was hypothesized that inhibition of ERK may have the same effect, which was also confirmed by the present study experiments. These findings showed that HuCCT1 and RBE cells treated with ERK inhibitor did not only reduce the expression of PD-L1, but also induced degradation of p62, conversion of LC3-I to LC3-II, and increase in LC3 puncta in the cytoplasm, which indicated the autophagy activation. In line with the findings that autophagy inhibition enhances PD-L1 expression in gastric cancer [12], the present research showed for the first time that ERK inhibition partially degraded PD-L1 via the autophagy pathway. Both pharmacologically inhibiting autophagy and genetically silencing *ATG7* partially reversed the decrease in PD-L1 expression caused by the ERK inhibitor (Fig. 3). Consequently, the promoted autophagic flux was part of the PD-L1 down-regulation mechanism due to ERK inhibition. However, restriction of autophagy alone by CQ or knocking down *ATG7* did not significantly alter the PD-L1 expression, which meant that the autophagy pathway, which takes place downstream of p-ERK, is involved in the regulation of PD-L1 expression. Nevertheless, whether the alteration of PD-L1 modulates autophagy after ERK inhibition needs further exploration.

Undoubtedly, PD-1/PD-L1-associated immune escape remains the principal cause of a number of patients’ failure to achieve a durable response to PD-1/PD-L1 blockade [33]. As an important mechanism for tumor cells to evade immune surveillance, PD-L1 expressed on tumor cells prevented T cells from effectively identifying and eradicating them [34]. The immunohistochemical analysis also showed that compared to para-tumor tissues, PD-L1 staining intensity was predominantly higher in tumor tissues. Due to the binding between PD-1 on T cells and PD-L1 on tumor cells, T cells infiltrating the tumor microenvironment, which are probably the most potent weapon for tumor elimination [35], were dysfunctional and exhausted [36], leading to a reduction in apoptosis of cytotoxic T cell-mediated tumor cells. In view of this point, our results indicate that upon MEKi or ERKi treatment, the level of PD-L1 expression was visibly down-regulated. More importantly, the apoptosis rate in HuCCT1 or RBE cells pretreated with ERKi was enhanced after co-culture with CD3/CD28-activated CD8^+^T cells compared to that in the control group, which was manifested by an increase in tumor cell apoptosis rate detected by the CCK-8 assay or flow cytometry. Based on these results, it is feasible that blocking ERK phosphorylation might abrogate PD-L1-mediated immunosuppression and promote the infiltration of functional cytotoxic T lymphocytes in solid tumors. Thus, considering the anti-tumor effect of ERK inhibitor and its regulatory role in tumor microenvironment, we reasoned that a combination treatment of ERK-targeted therapy and anti-PD-1/PD-L1 immunotherapy might block interactions between PD-1/PD-L1 pathway molecules more completely and restore CD8^+^T cell recognition in tumor cells to enhance the T cell-mediated immune response and anti-tumor activity. A recent study has also reported that the ERK pathway inhibitor in combination with anti–PD-1 monoclonal antibody suppresses tumor growth and improves survival in mice [37].

In summary, the present study elucidated a novel mechanism by which ERK signaling regulates the PD-L1 expression via the autophagy degradation pathway and enhances the T cell-mediated immune response. These findings shed light on the clinical application of ERK-targeted therapy together with anti-PD-1/PD-L1 immunotherapy, which represents a promising strategy for RAS-mutated iCCA treatment.

## Data Availability

All data generated or analyzed during this study are included in this published article.
